# Melatonin Enhanced the Tolerance of *Arabidopsis thaliana* to High Light Through Improving Anti-oxidative System and Photosynthesis

**DOI:** 10.3389/fpls.2021.752584

**Published:** 2021-10-07

**Authors:** Si-Jia Yang, Bo Huang, Yu-Qing Zhao, Di Hu, Tao Chen, Chun-Bang Ding, Yang-Er Chen, Shu Yuan, Ming Yuan

**Affiliations:** ^1^College of Life Science, Sichuan Agricultural University, Ya’an, China; ^2^College of Resources, Sichuan Agricultural University, Chengdu, China

**Keywords:** *Arabidopsis*, melatonin, high light, ROS, photosynthetic protein

## Abstract

Land plants live in a crisis-filled environment and the fluctuation of sunlight intensity often causes damage to photosynthetic apparatus. Phyto-melatonin is an effective bioactive molecule that helps plants to resist various biotic and abiotic stresses. In order to explore the role of melatonin under high light stress, we investigated the effects of melatonin on anti-oxidative system and photosynthesis of *Arabidopsis thaliana* under high light. Results showed that exogenous melatonin increased photosynthetic rate and protected photosynthetic proteins under high light. This was mainly owing to the fact that exogenous melatonin effectively decreased the accumulation of reactive oxygen species and protected integrity of membrane and photosynthetic pigments, and reduced cell death. Taken together, our study promoted more comprehensive understanding in the protective effects of exogenous melatonin under high light.

## Highlights

- Melatonin inhibited the burst of reactive oxygen species by regulating enzymatic and non-enzymatic antioxidant systems under high light.
- Melatonin improved the photosynthesis under high light through maintaining the integrity of photosynthetic apparatus.
- Exogenous melatonin functions partially through improving the accumulation of endogenous melatonin, but it could not completely compensate for the deficiency of endogenous melatonin.

## Introduction

Plants depend on sunlight absolutely as an overall energy source so that they develop multiple protein complexes to accomplish photosynthesis. These protein complexes include Photosystem II (PSII), Photosystem I (PSI), cytochrome *b_6_f* complex, and so on (Jarvis and Lopez-Juez, 2013). When light energy is insufficient, plants capture more light energy through changing the location of chloroplast (Salgado-Luarte and Gianoli, 2011). When the absorbed light energy of plants exceeds their demand, the photosynthetic complexes will be injured, leading to the decrease of photosynthetic efficiency (Demmig-adams and Adams, 1992). At the same time, reactive oxygen species (ROS) bursts (Jarvis and Lopez-Juez, 2013), and the resultant ROS is toxic to plants (Nishiyama et al., 2001). Although the damage mechanism of photosynthetic apparatus caused by high light is still controversial, it is indisputable that the high light finally leads to injure D1 subunit of PSII (Allakhverdiev and Murata, 2004). The photodamage of D1 occurs at all light intensities, but the photoinhibition occurs only when the balance between the photodamage and repair of D1 is broken (Allakhverdiev and Murata, 2004). Fortunately, plants had established an elaborate protective mechanism, including chloroplast avoidance movement which could minimize light exposure, ROS scavenging systems that are composed of SOD, POD, APX, etc. (Apel and Hirt, 2004), and PSII repair cycle (Kirchhoff, 2014). Although this multi-level photoprotective mechanism helps plants to minimize the injury on the photosynthetic machinery, the damage is unavoidable. Even the damage would affect plant growth and development, resulting in yield reduction and death.

Melatonin (N-acetyl-5-methoxytryptamine), a kind of indoleamine which widely exists in organism, was discovered in plants in 1995 and numerous studies proved that melatonin has involved in multiple processes in plants, including the development of flower (Lee et al., 2019), the architecture of root (Yang et al., 2021), the ripening of fruit (Wang et al., 2020), the senescence of leaf (Wang et al., 2013), the regulation of circadian rhythms, and the protective effect on chlorophyll and photosynthesis (Arnao and Hernandez-Ruiz, 2015). Melatonin alleviated oxidative damage through effectively scavenging ROS and reactive nitrogen species (RNS) (Arnao and Hernandez-Ruiz, 2015). And its metabolites, such as 2-hydroxylmelatonin and N1-acetyl-N2-formyl-5-methoxykynuramine, could also directly and efficiently scavenge ROS (Tan et al., 2007). Besides, melatonin also inspired antioxidant activity by stimulating antioxidant enzymes and could augment the ascorbate-glutathione (AsA-GSH) cycle to scavenge excess ROS (Li et al., 2015). And melatonin helped plants to defend against multiple abiotic stresses, such as cold, heavy metals, salt, drought, and so on (Arnao and Hernandez-Ruiz, 2015). Exogenous melatonin relieved the photoinhibition of tomato seedlings by improving non-photochemical quenching under cold stress (Ding et al., 2017). Similarly, the accumulation of melatonin in water hyacinth under sunlight was significantly higher than that under artificial low-light (Tan et al., 2007). This implies that melatonin can be induced by high light. Supporting these results, the expression of the melatonin-synthesis-related gene *ASMT* in apple had been up-regulated by high light, leading to the accumulation of melatonin (Zheng et al., 2017). In addition, melatonin enhanced the tolerance to high light in *Arabidopsis thaliana* (Lee and Back, 2018). However, the underlying physiological and molecular mechanism of the elevated tolerance to high light by melatonin remains unclear in plants.

Plants need light for photosynthesis and thus gain energy for their growth, but excessively high light does harm to photosynthetic apparatus. There were many researches on the high light stress in plants, but the role of melatonin under high light had been less explored. Lee and Back (2018) found that high light led to the brust of ROS, and the synthesis of melatonin was induced by chloroplastidic singlet oxygen and promoted the accumulation of melatonin. At the same time, melatonin increased the activity of antioxidant enzymes, thus enhancing the tolerance of plants to high light. In addition, Yao et al. (2020) reported the synthesis of melatonin was induced by UV-B. The wavelength of light spectrum also affected the synthesis of melatonin. Afreen et al. (2006) reported that the melatonin concentrations were highest in red-light-exposed plants and followed the blue light and white light. A lot of study showed that high light inhibited photosynthesis, but the role of melatonin in this physiological process is still unclosed.

Based on the reported relationship between melatonin and light intensity, we suggested that melatonin decreased the level of ROS by regulating antioxidant system to protect the photosynthesis under high light. To test this hypothesis, we measured ROS accumulation, membrane lipid peroxide, photosynthetic parameters, antioxidant enzyme, and PSII protein after the melatonin pretreatment under high light. The results demonstrated that melatonin provided effective ROS scavenging ability for plants and preserved the integrity of the photosynthetic protein, and then enhanced the tolerance to high light.

## Materials and Methods

### Plant Materials and Treatments

*Arabidopsis thaliana*, including wild-type (Col-0) and mutants, were grown in pots filled with the mixture of humus, perlite, and vermiculite at the ratio of 1:1:1 with 60% relative humidity and illumination of 120μmolm^−2^ s^−1^ for a 16h (22°C)/8h (20°C) day/night photoperiod. SALK_032239 (*SNAT-1*) and SALK_020577 (*SNAT-2*) were obtained from the Arabidopsis Biological Resource Center (Ohio State University, Columbus, OH, United States). Arabidopsis leaves were sprayed 100μmol/L melatonin (with 0.02% Tween-20) on the 26th day, and sprayed again after 24h. Then, the seedlings were exposed to high light (1,000μmolm^−2^ s^−1^) for 3h. All experiments were performed in triplicate.

### Determination of Chlorophyll and Carotenoid Content

Chlorophyll (Chl) and carotenoid was determined by the previously described method (Lichtenthaler and Wellburn, 1983). Fresh leaves (0.1g) were cut and homogenized with 5ml of 80% (v/v) acetone, then centrifuged at 8,000 r min^−1^ for 10min. The absorbance of the supernatant was recorded with a spectrophotometer (UV-1750, Shimadzu, Japan) at 663, 646 and 470nm.

### Melatonin Measurement

Extraction of melatonin from *Arabidopsis* was performed as described by Han et al. (2017). The quantification of melatonin was performed with liquid a chromatography (HPLC) system (1290 LC, Agilent, United States) couple to a mass spectrum (MS) system (6470 LC-MS/MS, Agilent, United States) according to Han et al. (2017). Separations were carried out on a 150×2.1mm, 1.8μm, Eldath RS-C18 column. Solvent A was methanol, and solvent B was methanol with 0.1% formic acid, v/v. The injection volume was 1μL, and solvent A was from 20 to 80% at a flow rate of 0.3mLmin^−1^. Mass spectrum parameters were as follows: positive ion mode; turbo 1 speed, 100%; turbo 2 speed, 100%; sheath gas temperature, 300°C; sheath gas flow, 11.0Lmin^−1^; capillary current, 59nA; capillary, 3,368V; MS 1 heater, 100°C; MS 2 heater, 100°C; rough vac, 9.91E-1Torr; high vac, 3.60E-5Torr; and m/z, 159.0.

### Measurement of Photosynthetic Characteristics and Chlorophyll Fluorescence

The photosynthetic rate (*P_n_*) and stomatal conductance (*g_s_*) of leaves was measured with a potable photosynthesis system (GSF-3000, Heinz-Walz Instruments, Effeltrich, Germany). Intact leaves were measured at a temperature of 22°C, the light intensity of 120μmolm^−2^ s^−1^ and 1,000μmolm^−2^ s^−1^, photosynthetically active radiation (PAR) of 750μmolm^−2^ s^−1^, the relative humidity of 65% (Huang et al., 2019).

Chlorophyll fluorescence was imaged with a modulated imaging fluorometer (the Imaging PAM M-Series Chlorophyll Fluorescence System, Heinz Walz Instruments, Effeltrich, Germany). The maximum efficiency of PSII photochemistry (Fv/Fm) and non-photochemical quenching (NPQ) was imaged and calculated after adaption in the dark for 30min (Huang et al., 2019).

### Determination of H_2_O_2_ and $O_{2}^{-}$

Histochemical detection of ROS was conducted as described by Han et al. (2017) Briefly, hydrogen peroxide (H_2_O_2_) and superoxide anion radicals ($O_{2}^{-}$) were visually detected with 0.5mg/ml 3,3′-diaminobenzidine (DAB) and 1mg/ml nitro blue tetrazolium (NBT), respectively. Then, the tissues were decolorized for 2h in boiling ethanol (85%). The quantification of H_2_O_2_ and $O_{2}^{-}$ was determined as described by Han et al. (2017).

### Determination of EL and MDA

Electrolyte leakage (EL) of leaves was measured with a conductivity meter (DDS-309+, Chengdu, China) as described by Han et al. (2017) The relative EL was obtained according to the ratio of the initial conductivity to the absolute conductivity. The degree of membrane lipid peroxidation in leaves was estimated by malondialdehyde (MDA) content. MDA was evaluated using thiobarbituric acid assay (Han et al., 2017).

### Trypan Blue Staining

The method of trypan blue dyeing according to Liang et al. (2015). Leaves were detached and stained with lactophenol-trypan blue solution (10ml of lactic acid, 10ml of glycerol, 10g of phenol, 10mg of trypan blue, dissolved in 10ml of distilled water) at 70°C for 1h and then boiled for approximately 5min and left staining overnight. After destaining in chloral hydrate solution (2.5g of chloral hydrate dissolved in 1ml of distilled water) for 3days to reduce background staining, samples were equilibrated with 70% glycerol for scanning.

### Assay of Antioxidant Enzymes and Non-enzymatic Antioxidant

For determination of SOD, POD, APX and GPX activities, the sample (0.5g) was homogenized in 5ml pre-cooled extract solution (50mm potassium phosphate buffer, pH 7.8). The homogenate was centrifuged for 20min at 12,000 r min^−1^ at 4°C, and the supernatant was used for further analysis.

The supernatant was used for assays of specific enzymatic activities. The activity of SOD (EC 1.15.1.1) was assessed according to Han et al. (2017) by measuring its ability to inhibit the photochemical reduction of NBT. One unit of SOD activity was defined as the amount of enzyme that caused 50% inhibition of NBT reduction. The activities of antioxidant enzymes, namely peroxidase (POD, EC 1.11.1.7), glutathione peroxidase (GPX, EC 1.11.1.9) and ascorbate peroxidase (APX, EC 1.11.1.11), were assayed following the methods of Huang et al. (2019).

The antioxidants including reduced ascorbic acid (AsA), dehydroascorbate (DHA), reduced glutathione (GSH) and oxidized glutathione (GSSG) were determined with the enzymatic cycling assay method (Han et al., 2017). For GSH, 0.5g sample was extracted in an ice bath with 5ml 100mm potassium phosphate buffer (pH 7.5) containing 5mm EDTA. After centrifugation, 2ml supernatant was mixed with 1ml 100mm phosphate buffer (pH 7.5) and 0.5ml 4mm DTNB (5,5′-dithio-bisnitrobenzoic acid). The reaction mixture was incubated at 25°C for 10min, and the absorbance at 412nm was measured. For the GSSG assay, the GSH in the supernatant was cleared first, and GSSG content was quantified as described by Han et al. (2017). The GSH and GSSG content was calculated according to their standard curves and expressed as μmol g^−1^(FW).

### Thylakoid Protein Analysis

Thylakoid membrane protein was isolated as described by Fristedt et al. (2010). Western blotting was performed according to Chen et al. (2009). The first antibody was PSII D1, D2, CP43, PsbS, Lhcb1, Lhcb2, Lhcb3, Lhcb4, Lhcb5, Lhcb6, and PSI PsaD, Lhca1, Lhca2, Lhca3 polyclonal antibody (Agrisera, Umea, Sweden), and the second antibody was goat anti-rabbit-HR (horseradish peroxidase; Agrisera, Umea, Sweden).

### Data Analysis

All experiments were repeated at least three times, and all data are presented as mean±standard deviation. Statistical analysis was done with IBM SPSS Statistics 20.0 software (IBM Corp., Armonk, NY, United States). Asterisks indicate significantly different values at ^*^p<0.05.

## Result

### Application of Exogenous Melatonin Enhanced the Accumulation of Melatonin in Leaf Tissue

**Figure 1:**
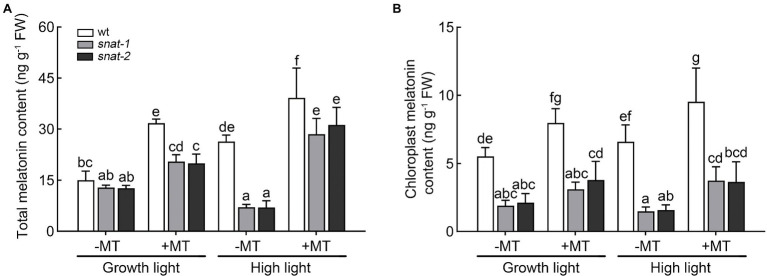
Effects of exogenous melatonin on the content of melatonin. **(A)** The melatonin content in leaves. **(B)** The melatonin content in chloroplasts. Data represent means ± SDs of three replicate samples. Different letters denote significant differences (Tukey’s HSD *post hoc* test; *p*<0.05).

After melatonin pretreatment, the level of melatonin in wild type increased 111.25%, and that in *snat-1* and *snat-2* increased 59.32 and 57.75%, respectively (Figure 1). Exogenous melatonin increased the content of melatonin in wild-type chloroplasts, but had no significant effect on the mutants (*snat-1*, *snat-2*). In addition, high light increased the level of melatonin in the wild type, but not in *snat-1* and *snat-2*. The application of melatonin further increase the level of melatonin in leaf tissue and chloroplast under high light. The above results suggested that exogenous melatonin could increase the content of melatonin by absorption and transport, and also might promote the synthesis of melatonin. Furthermore, high light could promote the synthesis of melatonin.

### Melatonin Protected Photosynthetic Pigments Under High Light

**Figure 2:**
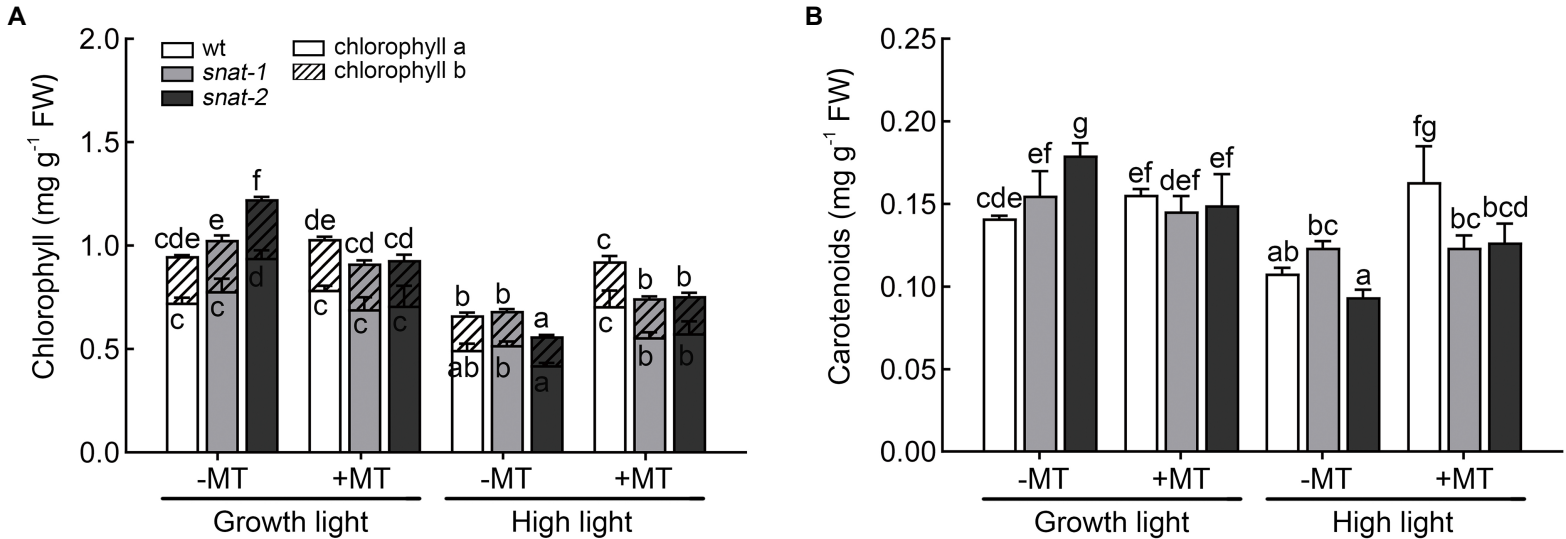
Effects of exogenous melatonin on photosynthetic pigments. **(A)** The chlorophyll level. **(B)** The carotenoid level. Others are the same as in Figure 1.

High light caused *Arabidopsis* leaves curling and chlorosis while melatonin pretreatment alleviated this symptom to a certain extent (Supplementary Figure S1A). But the application of melatonin had no effect on the fresh weight and dry weight of *seedlings* (Supplementary Figures S1B,C). The level of chlorophyll and carotenoid significantly decreased under high light, but this situation was significantly ameliorated after melatonin pretreatment (Figure 2). But the level of chlorophyll and carotenoid in *snat-1* and *snat-2* was still lower than that in the wild type after melatonin pretreatment under high light. This indicated that the lack of endogenous melatonin could influence the rescue of chlorophyll and carotenoid by exogenous melatonin under high light.

### Melatonin Protected Photosynthesis Under High Light

**Figure 3:**
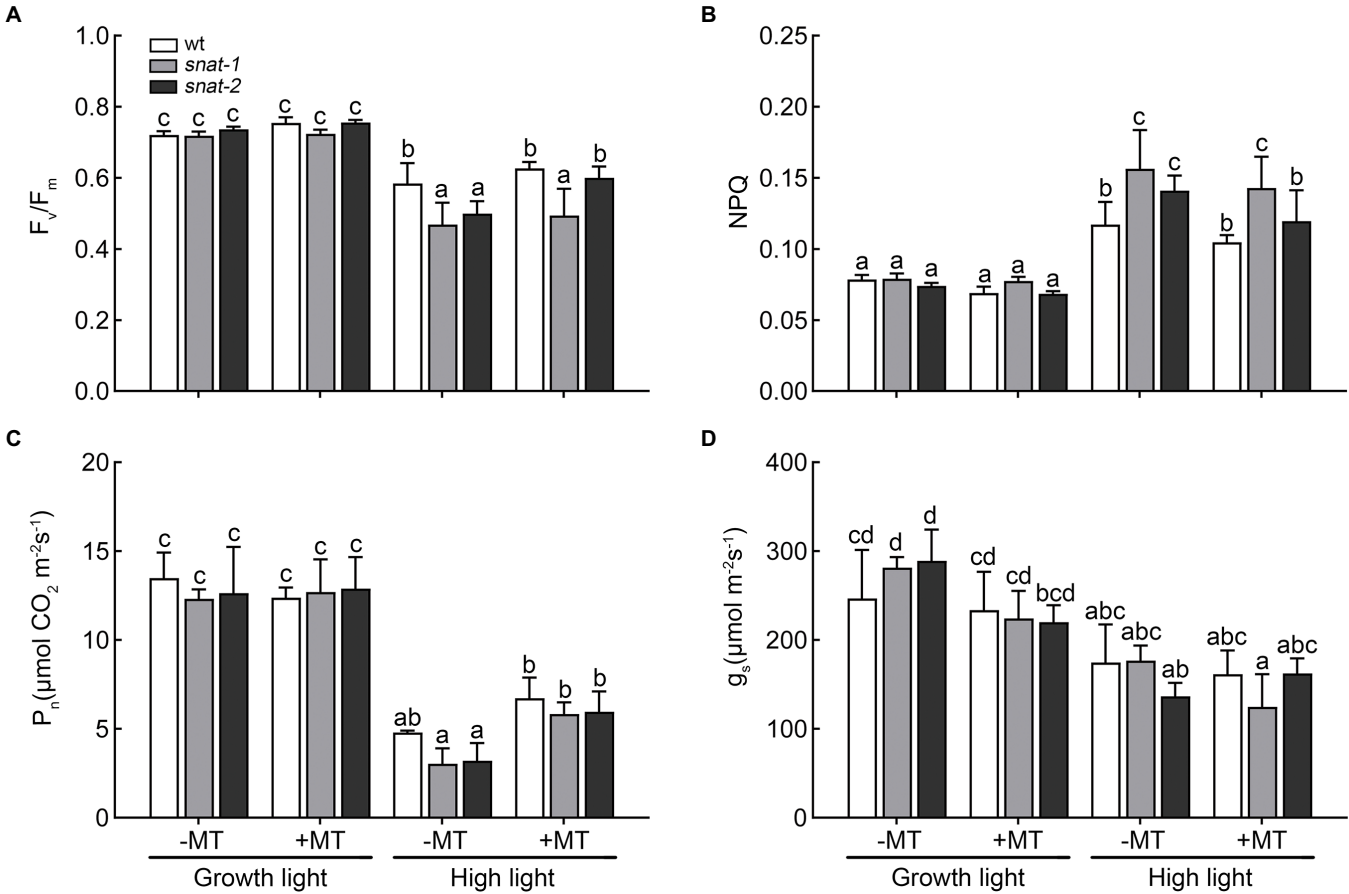
Effects of exogenous melatonin on photosynthesis. **(A)** Maximum PSII quantum yield (F_v_/F_m_). **(B)** Non-photochemical quenching (NPQ). **(C)** Net photosynthetic rate (*P_n_*). **(D)** Stomatal conductance (*g_s_*). Others are the same as in Figure 1.

Under growth light, exogenous melatonin had little influence on photosynthesis. The Fv/Fm of *snat-1* and *snat-2* decreased more than WT under high light, but they all recovered after melatonin pretreatment (Figure 3A). NPQ significantly increased after 3h high light, and the NPQ of the *snat-1* and *snat-2* were higher than WT (Figure 3B). High light significantly decreased *P_n_* and *g_s_* in both WT and mutants, and mutants showed a larger drop (Figures 3C,D). Exogenous melatonin increased *P_n_* under high light, but showed no effects on *g_s_*. These results showed that high light could cause obvious damage to the photosynthesis and reduce photosynthetic efficiency, but melatonin could reverse this trend.

### Melatonin Decreased the Level of ROS and Reduced the Damage to Cell

**Figure 4:**
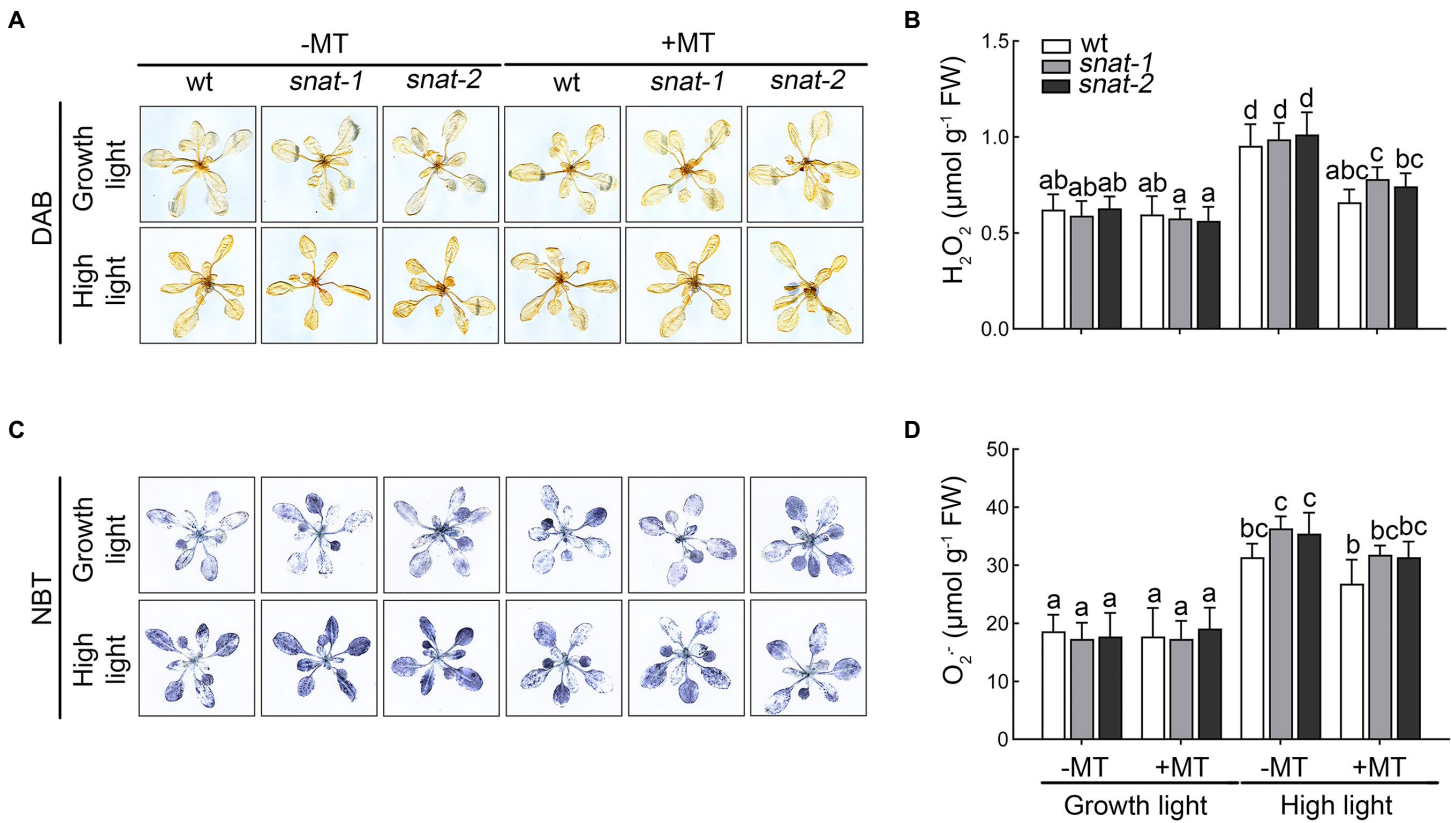
Effects of exogenous melatonin on reactive oxygen species. **(A)** Accumulation of hydrogen peroxide (H_2_O_2_) was visualized by 3,3′-diaminobenzidine (DAB) staining. **(B)** The content of hydrogen peroxide (H_2_O_2_). **(C)** Accumulation of superoxide anion radicals ($O_{2}^{-}$) was visualized by nitrotetrazolium blue (NBT) staining. **(D)** The content of superoxide anion radicals ($O_{2}^{-}$).

H_2_O_2_ and $O_{2}^{-}$ are two major ROS which produced in chloroplasts under high light and caused oxidative damage to the photosystem. The histochemical staining and quantitative analysis showed high light promoted the production of H_2_O_2_ and $O_{2}^{-}$, and exogenous melatonin decreased their accumulation (Figure 4). The content of ROS in the chloroplast showed the same trend as that in leaves (Supplementary Figure S2). These results showed that high light caused the brust of ROS but exogenous melatonin relieved this dilemma.

**Figure 5:**
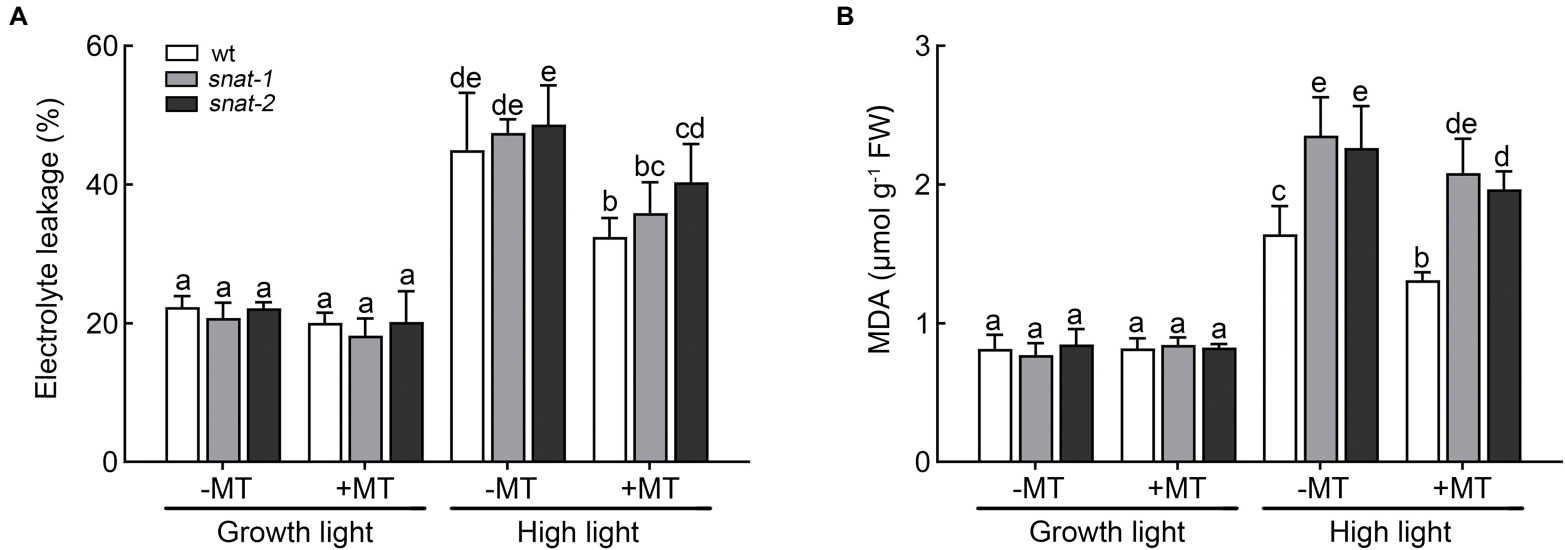
Effects of exogenous melatonin on the damage of cellular membranes. **(A)** Electrolyte leakage (EL). **(B)** Malondialdehyde (MDA). Others are the same as in Figure 1.

The levels of EL and MDA increased significantly under high light. Exogenous melatonin lessened the increase of EL and MDA, and the alleviation role in *snat-1* and *snat-2* was weaker than that in WT (Figures 5A,B).

**Figure 6:**
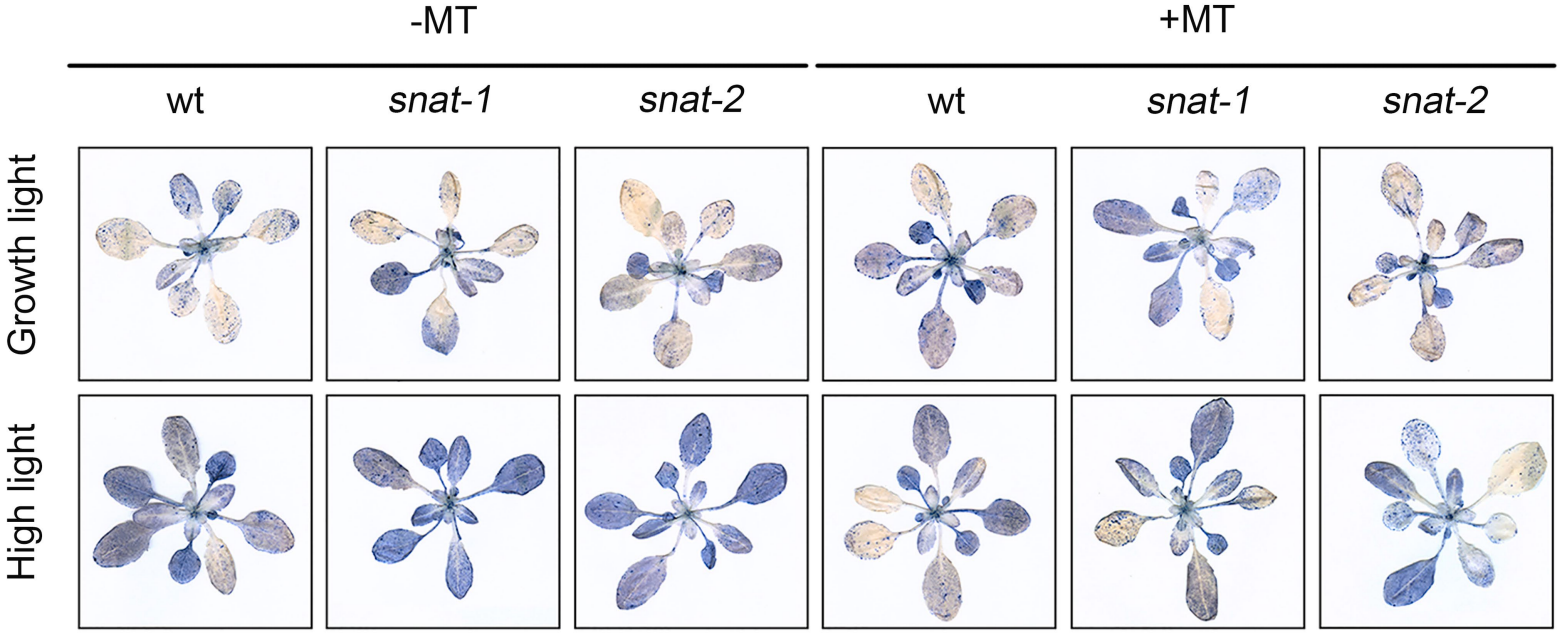
Effects of exogenous melatonin on cell death. Trypan-blue staining of the Col-0 and mutants (*snat-1*, *snat-2*) under the growth light and high light.

Cell death enhanced under high light, and it was even worse in *snat-1* and *snat-2*. Melatonin pretreatment reduced the level of cell death under high light, but it was still more serious in *snat-1* and *snat-2* (Figure 6).

These results showed that exogenous melatonin could alleviate the damage of cell membrane and inhibit cell death under high light. It is worth noting that exogenous melatonin could not completely compensate for the deficiency of endogenous melatonin.

### Melatonin Promoted the Antioxidant Ability in Plant Under High Light

The content of soluble sugar and proline increased under high light, and exogenous melatonin could downregulate the level of soluble sugar and proline (Supplementary Figure S3).

High light increased the content of AsA and GSH in WT, but there was no obvious effect on that of mutants (Table 1). Exogenous melatonin increased the content of AsA and GSH in *snat-1* and *snat-2*, but not in WT under high light. High light decreased the ratio of AsA/DHA, and the ratio in WT was higher than that of *snat-1* and *snat-2*. Exogenous melatonin reversed this trend.

**Table 1:** Effects of exogenous melatonin on non-enzymatic antioxidant.

		Growth light		High light	
		−MT	+MT	−MT	+MT
AsA	Col-0	2.130 ± 0.059^ab^	2.145 ± 0.057^ab^	2.469 ± 0.069^c^	2.451 ± 0.115^c^
	*snat-1*	2.234 ± 0.246^abc^	2.101 ± 0.094^a^	2.134 ± 0.069^ab^	2.445 ± 0.171^c^
	*snat-2*	2.263 ± 0.162^abc^	2.081 ± 0.060^a^	2.105 ± 0.062^a^	2.371 ± 0.222^bc^
DHA	Col-0	0.680 ± 0.062^a^	0.661 ± 0.071^a^	0.971 ± 0.033^bc^	0.917 ± 0.036^c^
	*snat-1*	0.736 ± 0.071^a^	0.663 ± 0.071^a^	1.101 ± 0.051^bc^	0.980 ± 0.116^bc^
	*snat-2*	0.685 ± 0.151^a^	0.647 ± 0.060^a^	0.969 ± 0.041^b^	1.000 ± 0.047^bc^
GSH	Col-0	0.454 ± 0.029^a^	0.458 ± 0.025^a^	0.556 ± 0.023^b^	0.564 ± 0.029^b^
	*snat-1*	0.429 ± 0.025^a^	0.455 ± 0.010^a^	0.460 ± 0.017^a^	0.521 ± 0.031^b^
	*snat-2*	0.453 ± 0.028^a^	0.450 ± 0.019^a^	0.463 ± 0.019^a^	0.551 ± 0.043^b^
GSSG	Col-0	0.146 ± 0.014^a^	0.144 ± 0.017^a^	0.245 ± 0.091^c^	0.184 ± 0.056^abc^
	*snat-1*	0.162 ± 0.017^ab^	0.161 ± 0.028^ab^	0.228 ± 0.036^bc^	0.240 ± 0.052^c^
	*snat-2*	0.154 ± 0.013^ab^	0.158 ± 0.040^ab^	0.218 ± 0.024^abc^	0.196 ± 0.017^abc^

*Different letters denote significant differences (Tukey’s HSD post hoc test; p<0.05)*.

**Figure 7:**
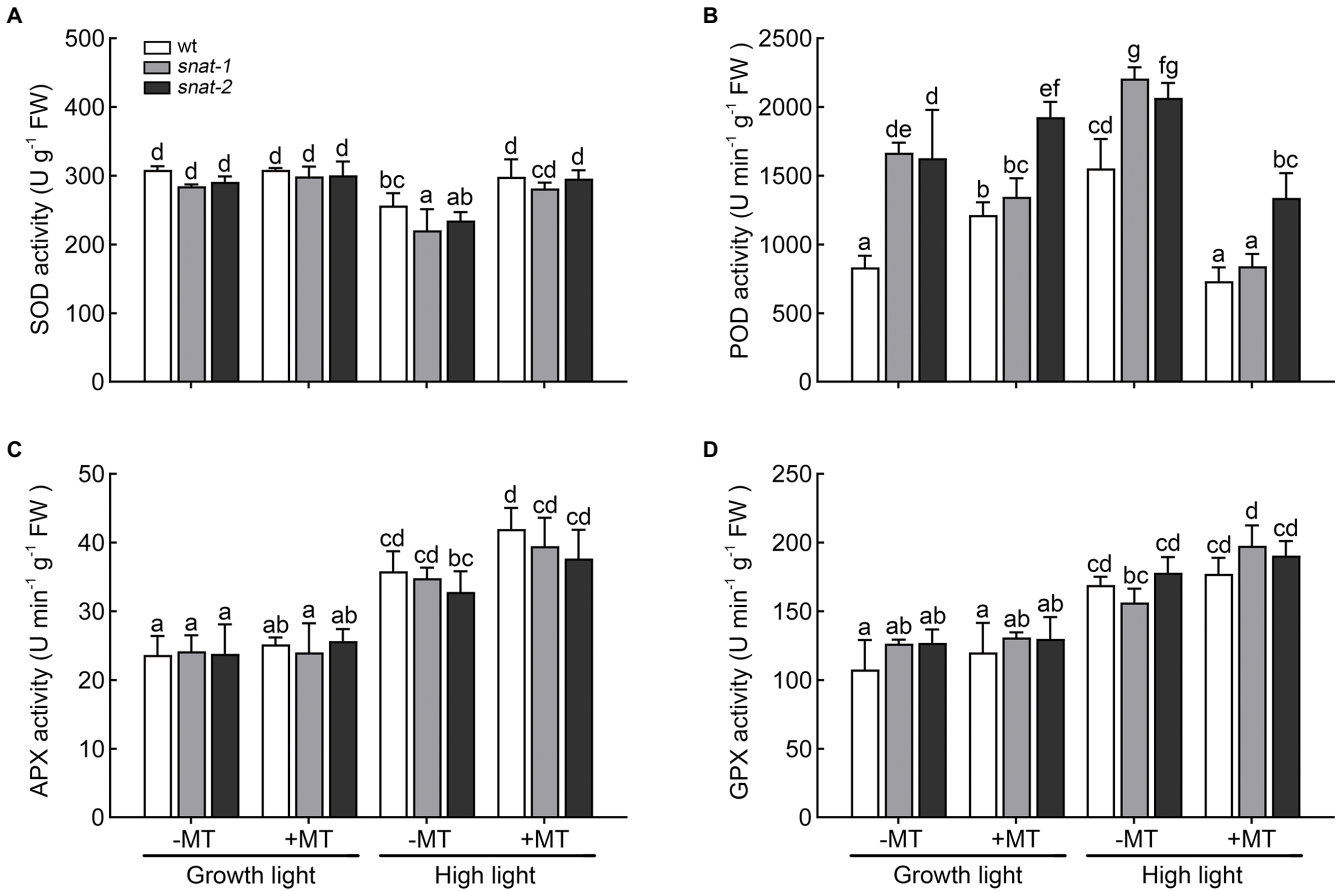
Effects of exogenous melatonin on the activity of antioxidant enzyme. **(A)** Superoxide dismutase (SOD) activity. **(B)** Peroxidase (POD) activity. **(C)** Ascorbate peroxidase (APX) activity. **(D)** Glutathione peroxidase (GPX) activity. Others are the same as in Figure 1.

Different antioxidant enzyme showed different response to high light and melatonin (Figure 7). The activities of POD, APX and GPX increased but SOD activity decreased under high light. Exogenous melatonin enhanced the activities of SOD, APX, and GPX but it decreased POD activity under high light.

On the one hand, melatonin directly removed ROS as a scavenger. On the other hand, it also regulated the level of non-enzymatic antioxidant and the activity of antioxidant enzymes. Therefore, melatonin works as a key regulator between antioxidants and ROS and contributes to the homeostasis of them.

### Exogenous Melatonin Protected Photosystem Protein Under High Light

**Figure 8:**
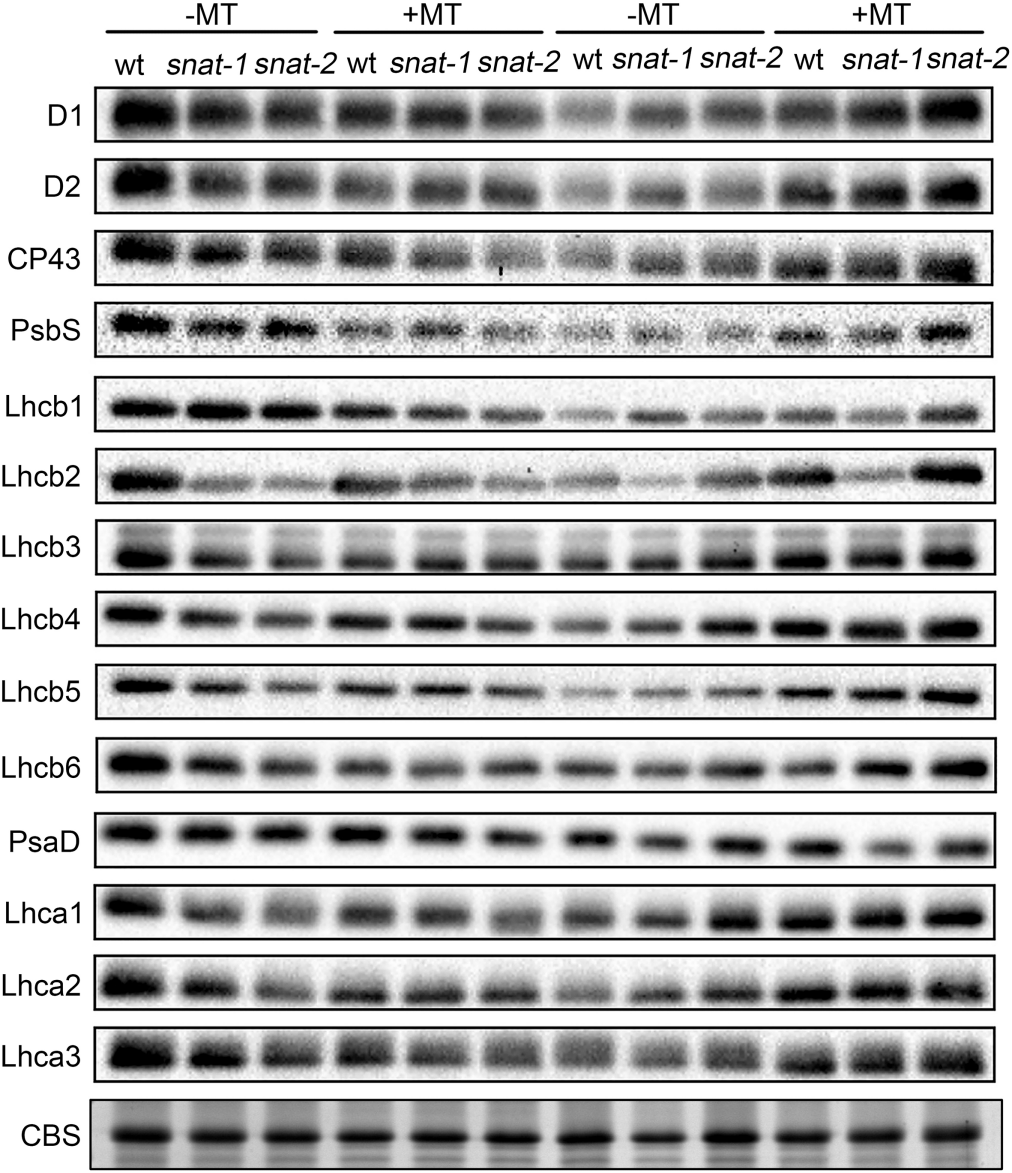
Effects of exogenous melatonin on thylakoid membrane proteins. Immunoblotting was performed with antibodies against D1, D2, CP43, PsbS, Lhcb1, Lhcb2, Lhcb3, Lhcb4, Lhcb5, Lhcb6, PsaD, Lhca1, Lhca2 and Lhca3. SDS-PAGE of thylakoid proteins stained with coomassie brilliant blue (CBS) worked as loading control.

Under growth light, the content of PSII proteins except Lhcb1 in *snat-1* and *snat-2* was lower than that in WT (Figure 8; Supplementary Figures 4, 5). Exogenous melatonin decreased the content of PSII proteins in WT and the content of PSII core proteins and Lhcb1 in *snat-1* and *snat-2*. High light decreased the content of PSII proteins in WT, and the content of PSII proteins except Lhcb2, Lhcb3, Lhcb4 in *snat-1* and *snat-2* also reduced. However, exogenous melatonin increased the content of PSII proteins except Lhcb6 in WT and the level of PSII proteins except Lhcb1 in *snat-1* and *snat-2* under high light.

Under growth light, the content of PSI proteins in *snat-1* and *snat-2* was lower than that of WT (Figure 8; Supplementary Figure 6). Exogenous melatonin decreased the level of PsaD, Lhca2 and Lhca3 in WT, and lowered the content of PsaD in *snat-1* and *snat-2*. High light decreased the content of PSI proteins in WT and *snat-1*. Exogenous melatonin improved the level of Lhca1, Lhca2 and Lhca3 under high light.

These results showed that melatonin reduced the level of photosystem proteins under growth light but it could alleviate the damage of photosystem proteins caused by high light.

## Discussion

Light is a source of energy and signal for plant growth. Plant has to go through a dark - low light - high light - low light - dark cycle every day. High light often causes destruction of the photosynthetic system and even cell death. Melatonin is a multitasking biomolecule, and it is involved in numerous physiological processes in plants, including redox reactions, biosynthesis, circadian clock, and stress defenses (Arnao and Hernandez-Ruiz, 2015). According to recent research, high light seriously destroyed the photosynthetic structure of chloroplasts and weakened its photosynthesis, and finally inhibited the growth of plants (Ding et al., 2017). In the present study, high light caused ROS burst and the reduction of photosynthesis. However, the application of exogenous melatonin significantly alleviated the damage caused by high light. Also, stress triggered endogenous melatonin response. Melatonin is effective in striving against stress, but the reception of stress signal, the activation of endogenous melatonin biosynthesis and the action process of melatonin were all restricted by many factors. At the cellular level, a stress signal is firstly received by the cell membrane, and then transferred to the nucleus. These starts to activate the melatonin biosynthesis pathway in mitochondria and chloroplasts by upregulating the melatonin-biosynthesis genes (Moustafa-Farag et al., 2020). Melatonin is effective in striving against stress, but the reception of stress signal, the activation of endogenous melatonin biosynthesis and the action process of melatonin were all restricted by many factors. In addition to ROS brust caused by high light to promote endogenous melatonin response, it seems that there are other pathways to promote the synthesis of melatonin. Transcription factors like MYB, bHLH, bZIP, ERF, NAC, and WRKY are major players in stress signaling and some constitute major hubs in the signaling webs (Tripathi et al., 2014). Hu et al. (2019) reported that high light up-regulated constantly the expression of 29 transcriptional factors, which could regulate the expression of genes associated with photosynthesis and ROS scavenging-related genes. Zhao et al. (2010) found that UV-B radiation induced *OsWRKY89* to participate in light responses. Transcription factor regulatory networks are also involved in the regulation of melatonin synthesis. Wei et al. (2017) reported that MeWRKY79 and MeHsf20 of cassava could act with W-box and thermal stress element HSEs (Heat-stress elements) in the promoter of *MeASMT* to induce its expression. And previous research found one cysteine2/histidine2-type zinc finger transcription factor, ZAT6, was involved in melatonin-mediated stress response in *Arabidopsis* (Shi and Chan, 2014). Maybe melatonin participated in light response through some of these transcription factors. In addition, transcription factor could directly affect the expression of melatonin synthesis gene, and also indirectly affect the effect on antioxidant system.

Reactive oxygen species is the byproducts of photosynthesis, respiration, and other normal metabolism, and it plays an important role in the resistance and tolerance to stress (Chen et al., 2016). Excessive ROS was toxic to cells and organisms, and involved in the programmed response to abiotic stress (Manchester et al., 2015). Yuan et al. (2020) found that high light triggered the accumulation of ROS. Han et al. (2017) found that exogenous melatonin decreased the level of ROS under cold stress. In the present study, H_2_O_2_ and $O_{2}^{-}$ increased significantly under high light, and they decreased with melatonin pretreatment.

Excessive ROS could stimulate membrane lipid peroxidation, and then led to the damage of cell membrane, loss of cellular integrity, and cell death (Chen et al., 2018). MDA and EL are regarded as important indicators of oxidative damage and previous study indicated that melatonin decreased the high level of EL and MDA caused by water stress (Zhang et al., 2013). Consistently, our research suggested that exogenous melatonin decreased the level of EL and MDA and reduced cell death under high light. It is worthwhile to note that the situation of *snat-1* and *snat-2* was worse than WT under high light. Exogenous melatonin application maintained a relatively low level of ROS and reduced the degree of cell damage, further conferring plant resistance to high light.

Enzymatic antioxidant system and non-enzymatic antioxidant system were evolved in response to oxidative stress in plants (Apel and Hirt, 2004). Melatonin alleviated oxidative damage caused by salinity, drought and cold perhaps by directly enhancing antioxidative enzyme activities, like SOD, POD and APX (Apel and Hirt, 2004). Chen et al. (2018) also found that the application of melatonin increased the activities of antioxidant enzymes in maize seedlings under salt stress. In the present research, melatonin increased the activity of SOD under high light. Exogenous melatonin downregulated the activity of POD and upregulated the activity of APX and GPX. They are all the converter for H_2_O_2_ but work in different ways. Melatonin inhibited the pathway of POD but promoted the pathway of APX and GPX to scavenge H_2_O_2_. Previous works showed that exogenous melatonin resulted in higher content of AsA and GSH under salt stress (Chen et al., 2018). But our study suggested that exogenous melatonin had little effect on them and the lack of endogenous melatonin weakened their levels. AsA-GSH cycle is a vital antioxidant system against oxidative stress in plants (Zhang et al., 2015). APX and GPX are the key enzymes of the glutathione ascorbic acid cycle, and melatonin effectively increased their activity. The glutathione-ascorbic acid cycle might play a key role in alleviating the high light stress. Melatonin did not only remove ROS as a scavenger but also regulated the activity of antioxidants in plants. Our results showed that melatonin reduced the accumulation of ROS but decreased the activity of POD. So melatonin was not overkill to ROS. In addition, Li et al. (2020) found that low concentration of melatonin induced the production of ROS and ROS worked as a key signal in many physiological processes. Maybe melatonin not only improves the defense capabilities of plant, but also ensures the role of ROS as a message transmitter in stress depending on its regulation role to the antioxidant system.

Photosynthetic pigments are susceptible to environmental stress. Melatonin effectively alleviated the degradation of chlorophyll and carotenoid under stress and made it with a certain level. Wu et al. (2021) found that melatonin suppressed the activities of chlorophyll catabolic enzymes such as chlorophyllase (CLH), pheophytinase (PPH), pheophorbide a oxygenase (PAO) and down-regulated the expressions of *BoNYC1*, *BoNOL*, *BoCLH*, *BoPPH*, *BoPAO*, *BoRCCR* and *BoSGR1* which involved in chlorophyll catabolism. In addition, Jahan et al. (2020) found melatonin upregulated the expression of chlorophyll synthesis genes, i.e., POR, CAO, CHL G.

The decrease of photosynthesis efficiency in plants after being exposed to adverse environmental might be a key reason for the reduction of crop. The previous studies showed abiotic stress induced irreversible damage to PSII in tomato, oat seeds, *Ligustrum vicaryi* and maize seedlings thereby decreasing photosynthetic rate (Ding et al., 2017; Chen et al., 2018; Alyammahi and Gururani, 2020; Kanwar et al., 2020; Yuan et al., 2020). The decrease of stomatal conductance could result in a declined *P_n_* and reduced assimilation products, thus causing an inhibited growth and a lower yield (Rao and Chaitanya, 2016). In this research, the *P_n_* and *g_s_* reduced under high light, and exogenous melatonin increased *P_n_*. These findings were in line with the report of maize under salt stress (Chen et al., 2018). Hu et al. (2021) suggested that the reduction of Chl a may be one of the reasons for the decrease of *P_n_* in acid rain stressed barley plants. And this was similar with our results (Figures 2A, 3C). The increase of *P_n_* by melatonin under high light might be due to its protective effect on chlorophyll. The *g_s_* was mainly controlled by guard cell through regulating the opening and closing of stomata (Assmann, 1999; Vavasseur and Raghavendra, 2005). Erland et al. (2019) employed a novel technique, quantum dot nanoparticles, to visualize the location of melatonin and found melatonin-QD aggregated in guard cells. It is possible that melatonin exerted an effect on *g_s_* through this pathway.

In nature, as soon as there is light, it will cause photooxidative damage to photosynthetic apparatus and then photoinhibition is unavoidable (Allakhverdiev and Murata, 2004). The extent of photoinhibition depends on the balance between photodamage and the repairing cycle (Allakhverdiev and Murata, 2004). Melatonin had been found to protect PSII proteins from oxidative injuries (Han et al., 2017; Huang et al., 2019). In previous work, the protective role of melatonin was confirmed on photosynthetic proteins in maize and tomato under drought and high light stress (Ding et al., 2017; Huang et al., 2019). Among ROS, H_2_O_2_ in chloroplast is an important inhibitor of the Calvin cycle. It might inhibit the activities of enzymes possessing sulfhydryl groups and reduced the photosynthetic CO_2_ assimilation (Hancock et al., 2005). In addition, the photooxidative damage products (especially H_2_O_2_) firstly stimulated the apparent photoinhibition of PSII by inhibiting the repair of PSII instead of accelerating photodamage to PSII (Nishiyama et al., 2001). In present study, we found that the change in the content of H_2_O_2_ in the chloroplast showed that the photosynthetic system was suffering from huge oxidative pressure, the proteins of the photosystem were destroyed under high light, and this situation was relieved by exogenous melatonin. Therefore, these results suggested that melatonin significantly inhibited ROS burst under high light. Numerous works had indicated that D1 protein is the key target under environmental stress and the D1 protein remained a relatively high level with melatonin pretreatment in our study.

Taken together, our research evaluated the effect and mechanism of melatonin on *Arabidopsis* under high light. Melatonin effectively protected photosynthesis in response to high light. Melatonin mainly worked through two aspects. On the one hand, melatonin was involved in cellular REDOX regulation. Melatonin directly removed ROS as antioxidants (Zhao et al., 2021). At the same time, melatonin regulated the activity of antioxidant enzyme as a signal molecule (Zhao et al., 2021). Therefore, melatonin protected photosynthetic pigments and proteins through redox homeostasis, and contributed to photosynthesis. On the other hand, melatonin gathered in guard cells (Erland et al., 2019), and might participate in stomatal movement. Simultaneously, the role of endogenous melatonin in plants was indispensable for the responses of plants to stress.

Our findings provided the evidence for melatonin to relieve high light stress, and extended new uses for melatonin as a plant growth regulator. At the same time, endogenous melatonin played an important role to against stress, and its potential mechanism needs further study. Our results and other reports suggested that melatonin might also be involved in stomatal movement (Assmann, 1999; Vavasseur and Raghavendra, 2005; Erland et al., 2019), but the mechanism is still unclear. Given the key role of melatonin in tolerance against various abiotic stresses, it is of interest to explore the mechanism of melatonin in plant.

## Data Availability Statement

The original contributions presented in the study are included in the article/Supplementary Material, further inquiries can be directed to the corresponding author.

## Author Contributions

MY designed the experiments. S-JY, BH, Y-QZ, DH, TC, and C-BD performed the experiments and data analysis. S-JY and MY wrote the manuscript. All authors contributed to the article and approved the submitted version.

## Conflict of Interest

The authors declare that the research was conducted in the absence of any commercial or financial relationships that could be construed as a potential conflict of interest.

## Publisher’s Note

All claims expressed in this article are solely those of the authors and do not necessarily represent those of their affiliated organizations, or those of the publisher, the editors and the reviewers. Any product that may be evaluated in this article, or claim that may be made by its manufacturer, is not guaranteed or endorsed by the publisher.

## Abbreviations

PSII: photosystem II
PSI: photosystem I
ROS: reactive oxygen species
H_2_O_2_: hydrogen peroxide
$O_{2}^{-}$: superoxide anion radicals
EL: electrolyte leakage
MDA: malondialdehyde
SOD: superoxide dismutase
POD: peroxidase
APX: ascorbate peroxidase
GPX: glutathione peroxidase
