# Supplementary material for: Melatonin Enhanced the Tolerance of Arabidopsis thaliana to High Light Through Improving Anti-oxidative System and Photosynthesis
Source: Front Plant Sci. 2021 Oct 7;12:752584. doi: 10.3389/fpls.2021.752584 (PMC8529209; doi:10.3389/fpls.2021.752584)
Supplement: Supplementary file 1 [file Table_1.DOC]

Table 1 Effects of exogenous melatonin on non-enzymatic antioxidant.

|  |  | Growth light | | High light | |
| --- | --- | --- | --- | --- | --- |
|  |  | -MT | +MT | -MT | +MT |
| AsA | Col-0 | 2.130±0.059^ab^ | 2.145±0.057^ab^ | 2.469±0.069^c^ | 2.451±0.115^c^ |
|  | *snat-1* | 2.234±0.246^abc^ | 2.101±0.094^a^ | 2.134±0.069^ab^ | 2.445±0.171^c^ |
|  | *snat-2* | 2.263±0.162^abc^ | 2.081±0.060^a^ | 2.105±0.062^a^ | 2.371±0.222^bc^ |
|  |  |  |  |  |  |
| DHA | Col-0 | 0.680±0.062^a^ | 0.661±0.071^a^ | 0.971±0.033^bc^ | 0.917±0.036^c^ |
|  | *snat-1* | 0.736±0.071^a^ | 0.663±0.071^a^ | 1.101±0.051^bc^ | 0.980±0.116^bc^ |
|  | *snat-2* | 0.685±0.151^a^ | 0.647±0.060^a^ | 0.969±0.041^b^ | 1.000±0.047^bc^ |
|  |  |  |  |  |  |
| GSH | Col-0 | 0.454±0.029^a^ | 0.458±0.025^a^ | 0.556±0.023^b^ | 0.564±0.029^b^ |
|  | *snat-1* | 0.429±0.025^a^ | 0.455±0.010^a^ | 0.460±0.017^a^ | 0.521±0.031^b^ |
|  | *snat-2* | 0.453±0.028^a^ | 0.450±0.019^a^ | 0.463±0.019^a^ | 0.551±0.043^b^ |
|  |  |  |  |  |  |
| GSSG | Col-0 | 0.146±0.014^a^ | 0.144±0.017^a^ | 0.245±0.091^c^ | 0.184±0.056^abc^ |
|  | *snat-1* | 0.162±0.017^ab^ | 0.161±0.028^ab^ | 0.228±0.036^bc^ | 0.240±0.052^c^ |
|  | *snat-2* | 0.154±0.013^ab^ | 0.158±0.040^ab^ | 0.218±0.024^abc^ | 0.196±0.017^abc^ |
