# Supplementary material for: Melatonin Enhanced the Tolerance of Arabidopsis thaliana to High Light Through Improving Anti-oxidative System and Photosynthesis
Source: Front Plant Sci. 2021 Oct 7;12:752584. doi: 10.3389/fpls.2021.752584 (PMC8529209; doi:10.3389/fpls.2021.752584)
Supplement: Supplementary file 2 [file Data_Sheet_1.PDF]

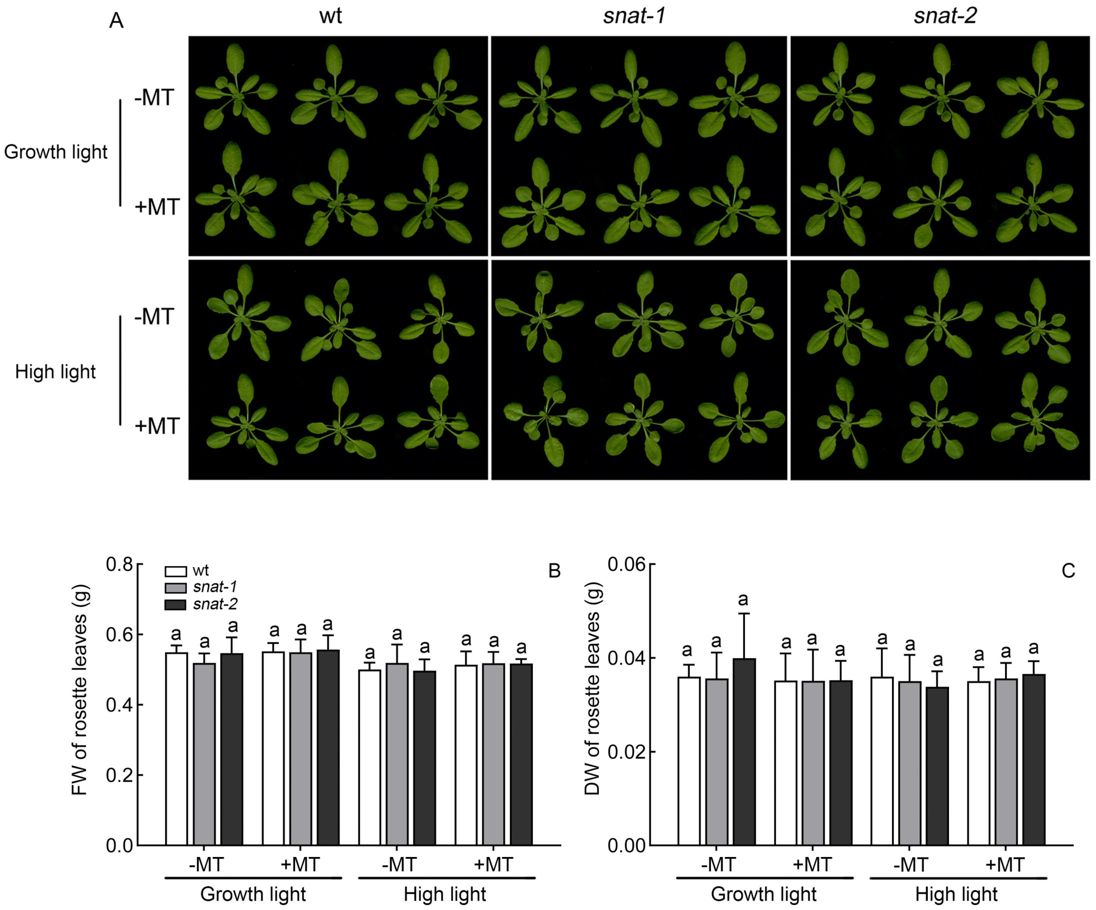

Supplemental Fig. 1 Effects of exogenous melatonin on phenotype and growth status of the Col-0 and mutants (*snat-1*, *snat-2*) under the GL and HL. (A) Phenotypes of Arabidopsis thaliana with melatonin pretreatment or not under growth light and high light stress. (B) Fresh weight of the Col-0 and mutants (*snat-1*, *snat-2*) after the above treatment. (C) Dry weight of the Col-0 and mutants (*snat-1*, *snat-2*) after the above treatment. Others are the same as in Figure 1.

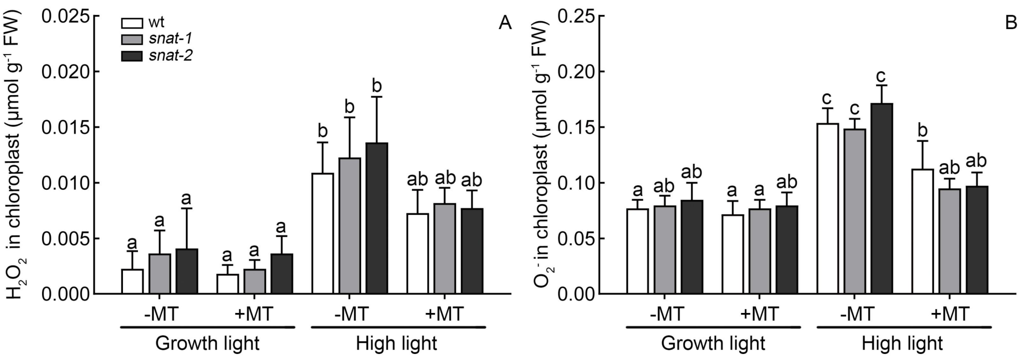

Supplemental Fig. 2 Effects of exogenous melatonin on reactive oxygen species of the Col-0 and mutants (*snat-1*, *snat-2*) under the GL and HL. (A) The content of H_2O_2 in chloroplast. (B) The content of O_2^- in chloroplast. Others are the same as in Figure 1.

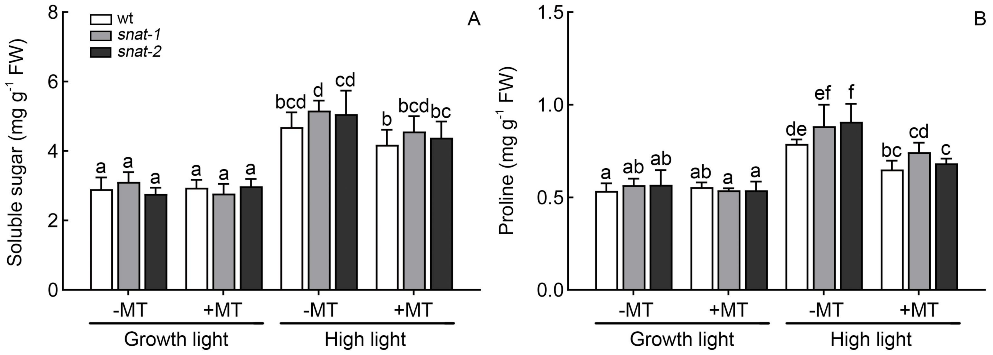

Supplemental Fig. 3 Effects of exogenous melatonin on cell damage situation. (A) Soluble sugar content. (B) Proline content. Others are the same as in Figure 1.

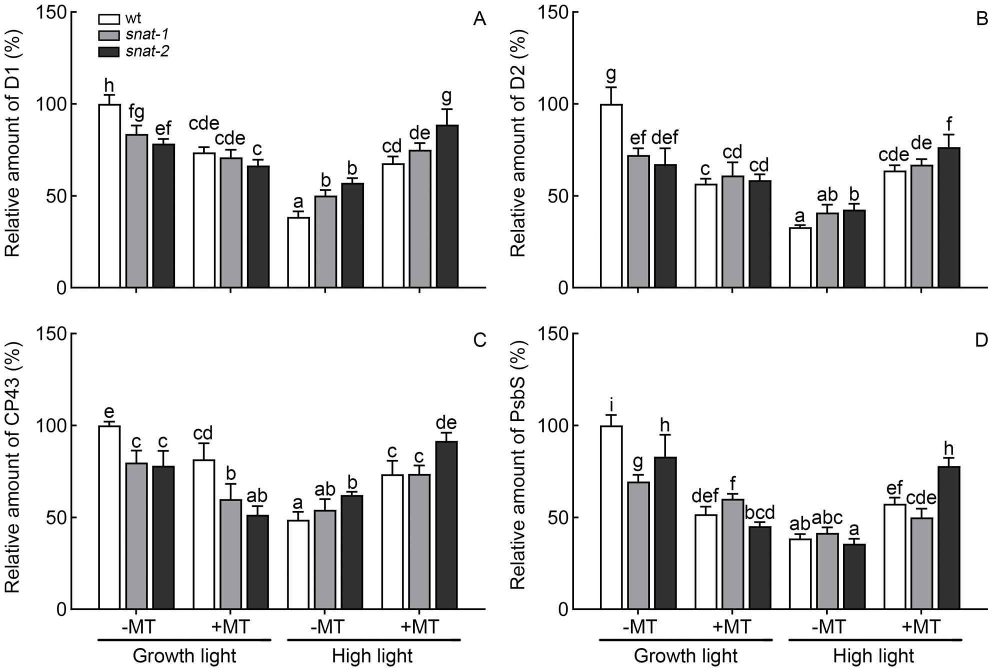

Supplemental Fig. 4 The quantification of immunoblot data, and results are relative to the amount of col-0 in the GL-MT (100%). Quantification of PSII core protein D1(A), D2(B), CP43(C) and PsbS(D). Others are the same as in Figure 1.

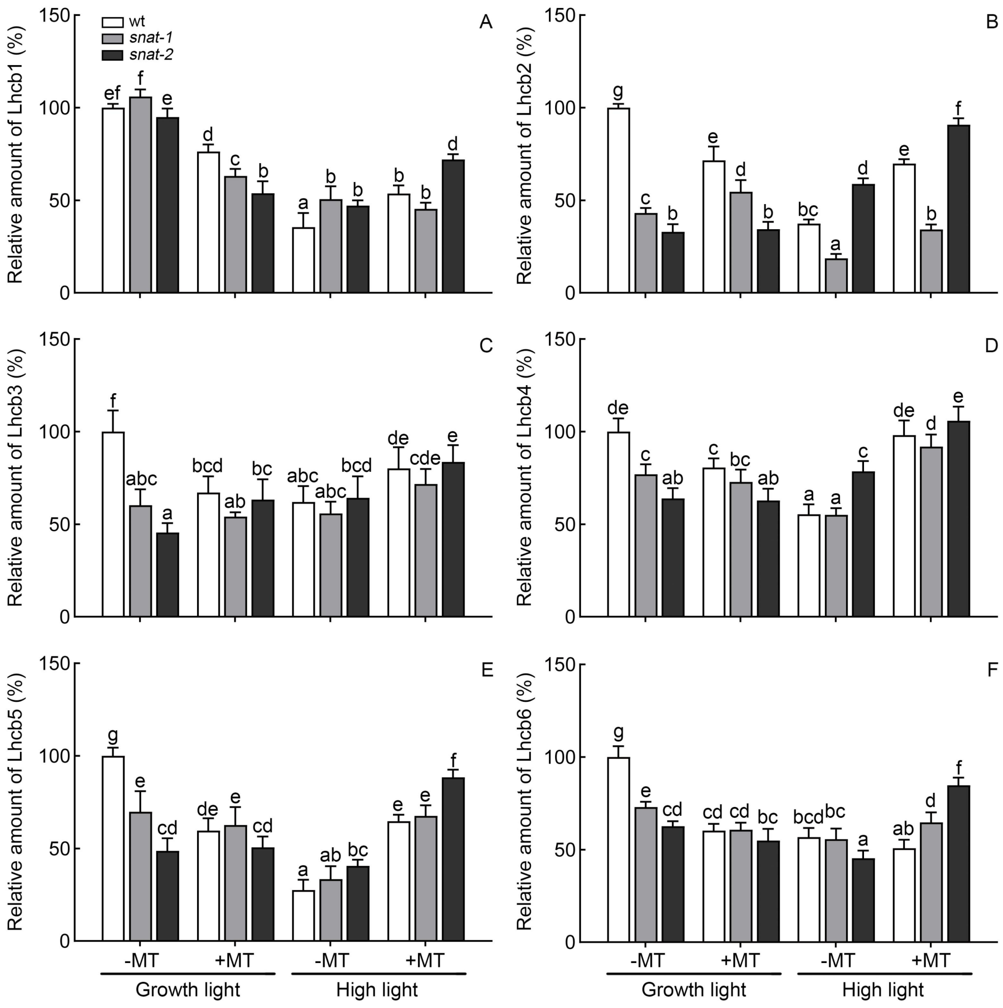

Supplemental Fig. 5 The quantification of immunoblot data, and results are relative to the amount of col-0 in the GL-MT (100%). Quantification of PSII light harvesting pigment protein Lhcb1(A), Lhcb2(B), Lhcb3(C), Lhcb4(D), Lhcb5(E) and Lhcb6(F). Others are the same as in Figure 1.

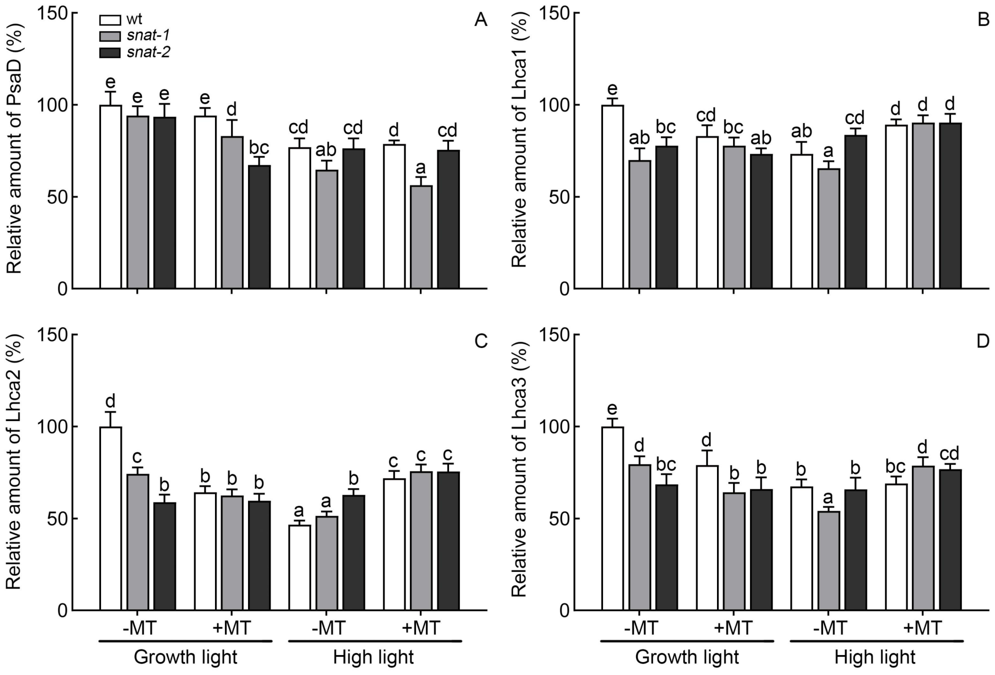

Supplemental Fig. 6 The quantification of immunoblot data, and results are relative to the amount of col-0 in the GL-MT (100%). Quantification of PSI core protein PsaD (A), light-harvesting complex proteins Lhca1(B), Lhca2 (C) and Lhca3(D). Others are the same as in Figure 1.
